# Response‐adapted zanubrutinib and tislelizumab as a potential strategy to enhance CD19 CAR T‐cell therapy in relapsed/refractory large B‐cell lymphoma: A retrospective observational study

**DOI:** 10.1002/ctm2.70310

**Published:** 2025-04-23

**Authors:** Rong Shen, Wei‐Guo Cao, Li Wang, Ling‐Shuang Sheng, Yi‐Lun Zhang, Wen Wu, Peng‐Peng Xu, Shu Cheng, Meng‐Ke Liu, Yan Dong, Yue Wang, Xiang‐Qin Weng, Xu‐Feng Jiang, Qi Song, Hong‐Mei Yi, Lei Li, Sheng Chen, Zi‐Xun Yan, Wei‐Li Zhao

**Affiliations:** ^1^ Shanghai Institute of Hematology, State Key Laboratory of Medical Genomics, National Research Center for Translational Medicine at Shanghai Ruijin Hospital affiliated to Shanghai Jiao Tong University School of Medicine Shanghai China; ^2^ Department of Radiation Oncology Ruijin Hospital affiliated to Shanghai Jiao Tong University School of Medicine Shanghai China; ^3^ Pôle de Recherches Sino‐Français en Science du Vivant et Génomique, Laboratory of Molecular Pathology Shanghai China; ^4^ Department of Nuclear Medicine Ruijin Hospital affiliated to Shanghai Jiao Tong University School of Medicine Shanghai China; ^5^ Department of Radiology Ruijin Hospital affiliated to Shanghai Jiao Tong University School of Medicine Shanghai China; ^6^ Department of Pathology Ruijin Hospital affiliated to Shanghai Jiao Tong University School of Medicine Shanghai China; ^7^ Department of Critical Care Medicine Ruijin Hospital affiliated to Shanghai Jiao Tong University School of Medicine Shanghai China; ^8^ Department of Neurology Ruijin Hospital affiliated to Shanghai Jiao Tong University School of Medicine Shanghai China

**Keywords:** bruton tyrosine kinase inhibitor, chimeric antigen receptor T‐cell therapy, large B‐cell lymphoma, programmed death‐1 inhibitor, tumour microenvironment

## Abstract

**Background:**

CD19 chimeric antigen receptor (CAR) T‐cell therapy is a potential treatment for relapsed/refractory (R/R) large B‐cell lymphoma (LBCL). The combination of targeted therapeutic strategies, particularly bruton tyrosine kinase inhibitor zanubrutinib and programmed death‐1 inhibitor tislelizumab, may improve clinical outcomes and modulate the tumour microenvironment (TME).

**Methods:**

We studied patients with R/R LBCL who received response‐adapted zanubrutinib plus tislelizumab upon CD19 CAR T‐cell therapy between June 2021 and March 2023. Patients were treated with zanubrutinib daily from leukapheresis to day 28 post‐infusion; those achieving complete response continued zanubrutinib monotherapy for 3 months, while partial responders received combined zanubrutinib for 3 months and tislelizumab for up to 2 years. We evaluated the overall response rate (ORR), complete response rate (CRR), progression‐free survival (PFS), overall survival (OS), and safety. DNA sequencing and RNA sequencing were performed on available tumour samples to analyse genetic aberrations and TME characteristics.

**Results:**

A total of 54 patients with LBCL were included, with a median follow‐up of 23.6 months. The ORR at day 28, month 3, and month 6 were 94% (CRR 66%), 87% (CRR 80%), and 80% (CRR 76%), respectively. The 2‐year PFS and 2‐year OS rates were 68% and 76%, respectively. Median PFS and median OS were not reached. Grade ≥ 3 cytokine release syndrome occurred in 9% of patients, with no grade ≥ 3 neurotoxicity observed. Genomic and transcriptomic data indicated that this regimen was effective across genetic subtypes and abrogated T‐cell exhaustion within the TME. However, tumour‐infiltrating M2 macrophages with dysregulated lipid metabolism were associated with poor clinical outcome.

**Conclusions:**

Response‐adapted zanubrutinib and tislelizumab potentially enhances the efficacy of CAR T‐cell therapy with a favourable safety profile in R/R LBCL, effectively counteracting T‐cell exhaustion. Future studies should focus on targeting M2 macrophages by reprogramming lipid metabolism to further attenuate the immunosuppressive TME.

**Highlights:**

- Response‐adapted zanubrutinib plus tislelizumab potentially enhances the efficacy of CAR T‐cell therapy for R/R LBCL with acceptable safety profile.
- This regimen functions independently of genetic subtypes, rendering it more applicable for clinical practice with CAR T‐cell therapy.
- This regimen effectively abrogates T‐cell exhaustion, but fails to overcome the immunosuppressive effects of M2 macrophages, providing a rationale for remodelling TME to optimise CAR T‐cell therapy.

## Graphical abstract

1. Response‐adapted zanubrutinib plus tislelizumab potentially enhances the efficacy of CAR T‐cell therapy for R/R LBCL with acceptable safety profile.
2. This regimen functions independently of genetic subtypes, rendering it more applicable for clinical practice with CAR T‐cell therapy.
3. This regimen effectively abrogates T‐cell exhaustion, but fails to overcome the immunosuppressive effects of M2 macrophages, providing a rationale for remodelling TME to optimise CAR T‐cell therapy.

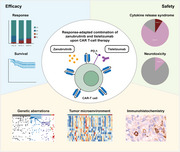

## 1 INTRODUCTION

CD19 chimeric antigen receptor (CAR)‐T cell therapy is a potential therapeutic approach for relapsed or refractory (R/R) large B‐cell lymphoma (LBCL).^1^ Currently, axicabtagene ciloleucel (axi‐cel) and relmacabtagene autoleucel (relma‐cel), which express CARs with a CD28 co‐stimulatory domain and a 4‐1BB co‐stimulatory domain, respectively, have been approved in China for treating R/R LBCL. Despite achieving a significant initial response following CAR T‐cell therapy, more than 50% of patients still experience rapid relapse and disease progression. Attaining complete response (CR) at 3 months is a predictive factor of long‐term response.^2^ More recently, growing evidence has suggested that the tumour microenvironment (TME) is of vital importance in lymphoma progression and relapse upon CAR T‐cell therapy. Tumour immune contexture features, such as exhausted T (Tex) cells highly expressing inhibitory receptors, as well as infiltration with immunosuppressive cells including M2 macrophages, regulatory T (Treg) cells, and myeloid‐derived suppressor cells (MDSCs), have been reported to influence the recruitment, expansion, and activity of CAR T‐cells, thereby contributing to resistance to CAR T‐cell therapy.^3^, ^4^

Bruton tyrosine kinase inhibitors (BTKi), initially developed to target B‐cell receptor signalling in tumour cells, have been reported to modulate the TME by altering the composition and functional profile of immune cells.^5^ Specifically, BTKi increase the abundance of CD4+ and CD8+ T cells, while reducing the expression of programmed death‐1 (PD‐1) and cytotoxic T‐lymphocyte‐associated antigen‐4 (CTLA‐4) in T cells.^6^ Combining immune checkpoint inhibitors (ICIs), particularly PD‐1 inhibitors (PD‐1i), with CAR T‐cell therapy has demonstrated encouraging results in preclinical studies and has increased CAR T‐cell counts in peripheral blood among treated patients.^7^, ^8^ The combination of zanubrutinib and CAR T‐cell therapy demonstrates notable efficacy and good tolerability in patients with R/R LBCL in some reports of clinical series.^9^, ^10^ However, how to perform response‐adapted treatment of BTKi plus PD‐1i remains great interests, aiming to synergise with CAR T‐cell therapy.

In this study, we investigated the effectiveness and tolerability of a response‐adapted regimen combining zanubrutinib and tislelizumab upon CD19 CAR T‐cell therapy in patients with R/R LBCL. Furthermore, we conducted genomic and transcriptomic analyses to identify biological factors related to the outcome of patients, providing a rationale for remodelling immunometabolism to optimise CAR T‐cell therapy.

## 2 METHODS

### 2.1 Study design

**FIGURE 1:**
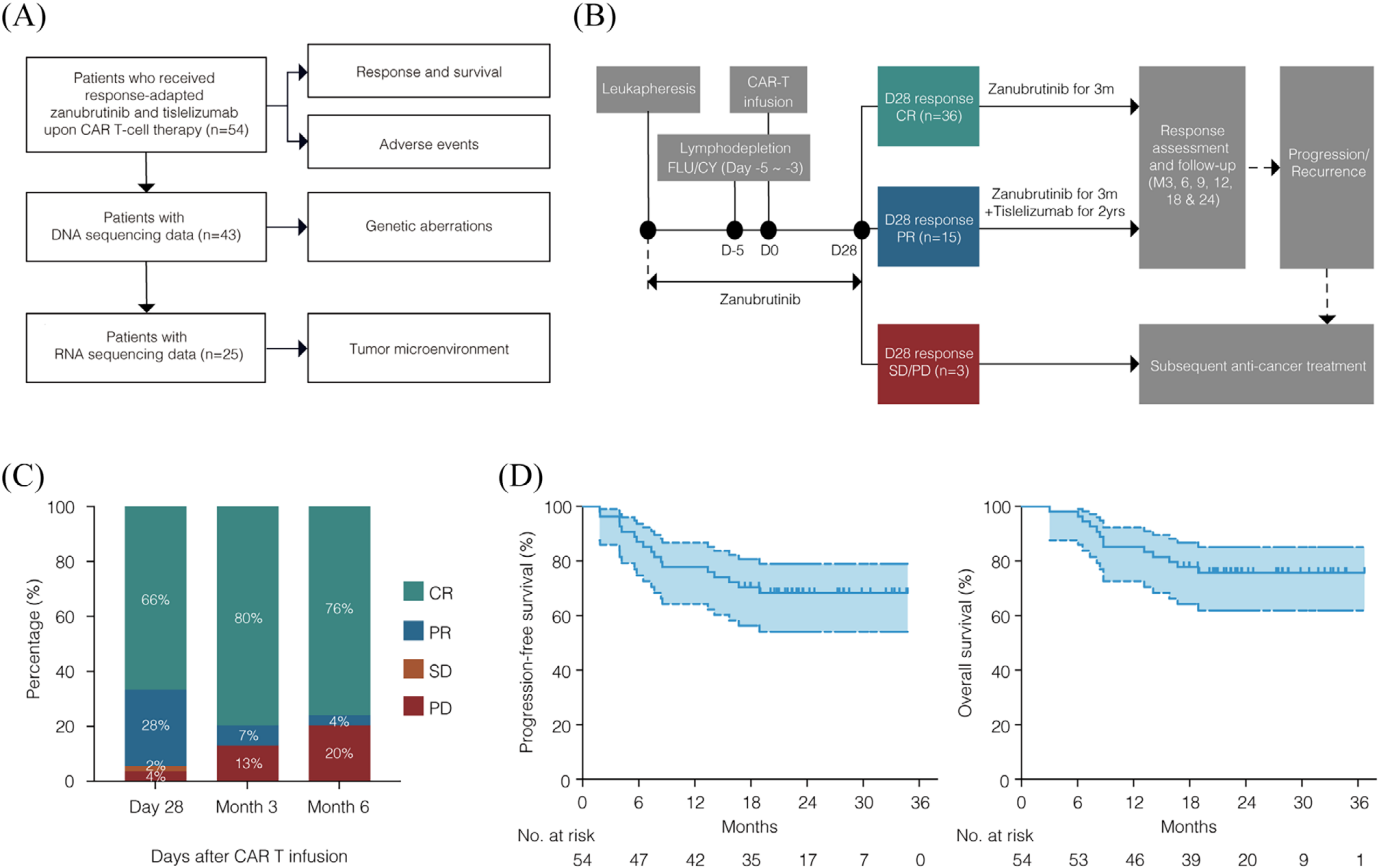
Outcome of patients treated with response‐adapted zanubrutinib plus tislelizumab upon CAR T‐cell therapy in R/R LBCL. (A) Flow diagram describing the population. A total of 54 patients were included. Before CAR T‐cell infusion, 43 patients had undergone genomic sequencing and 25 patients had undergone transcriptomic sequencing. (B) Schematic diagram of response‐adapted zanubrutinib and tislelizumab treatment upon CAR T‐cell therapy. Patients were treated with response‐adapted zanubrutinib plus tislelizumab upon CAR T‐cell therapy in the following procedure: patients received zanubrutinib 320 mg per day from leukapheresis to day 28 after CAR T‐cell infusion, then patients who attained CR at day 28 received zanubrutinib for 3 months, while those attained PR at day 28 received zanubrutinib for 3 months in combination with PD‐1 inhibitor tislelizumab (200 mg every 21 days) for 2 years. (C) Treatment response at day 28, month 3, and month 6 (*n* = 54). (D) PFS and OS of patients treated with response‐adapted zanubrutinib plus tislelizumab upon CAR T‐cell therapy.

This retrospective, single‐centre observational study included consecutive patients aged 18 years or older with histologically diagnosed CD19+ R/R LBCL who had previously received systemic treatment, including rituximab and anthracycline‐based chemotherapy. Patients received axicabtagene ciloleucel (axi‐cel) or relmacabtagene autoleucel (relma‐cel) at our centre between June 2021 and March 2023. The study was approved by the Shanghai Ruijin Hospital Review Board, and informed consent was obtained in accordance with the Declaration of Helsinki. All drugs used in this study, including axi‐cel, relma‐cel, zanubrutinib, and tislelizumab, were approved in China at the time of the study. The date of data cutoff for efficacy and safety analyses was 1 August 2024. Of the 54 patients included in this study, 44 were registered in the real‐world cohort of axi‐cel (ChiCTR2100047990) and 10 were registered in the real‐world cohort of relma‐cel (NCT06142175). DNA sequencing was performed on tumour samples from 43 patients, and RNA sequencing on samples from 25 patients (Figure 1A). Data relevant to CAR T‐cell therapy were obtained from medical records. Positron emission tomography‐computed tomography was used to measure the maximal diameter of the tumour mass.

CAR T‐cell therapy was delivered according to standard protocols. For patients who received axi‐cel, lymphodepleting chemotherapy was administered on days ‐5 through ‐3 with fludarabine (30 mg/m^2^ intravenous daily) and cyclophosphamide (500 mg/m^2^ intravenous daily). For those who received relma‐cel, lymphodepleting chemotherapy was administered on days ‐5 through ‐3 with fludarabine (25 mg/m^2^ intravenous daily) and cyclophosphamide (250 mg/m^2^ intravenous daily). The dose of fludarabine was adjusted according to the patients' creatinine clearance levels.

Patients were treated with response‐adapted zanubrutinib plus tislelizumab upon CAR T‐cell therapy in the following procedure. Zanubrutinib 320 mg daily was administered from leukapheresis through day 28 after CAR T‐cell infusion. Then patients achieving CR at day 28 continued on zanubrutinib monotherapy for 3 months, while those with PR received zanubrutinib for 3 months combined with tislelizumab 200 mg every 21 days for up to 2 years (Figure 1B). Patients received zanubrutinib or tislelizumab with appropriate dose modifications and interruptions. For grade 4 haematological adverse events (AEs), or grade ≥ 3 non‐haematological AEs, zanubrutinib was withheld until the AEs improved to grade 1, and then resumed at the original dose. For subsequent occurrences of AEs, the dose was adjusted to the next lower level. The use of hematopoietic growth factors was allowed. For grade 3 immune‐related adverse events (irAEs), tislelizumab was suspended until the irAEs resolved to grade 1 or below. If grade 4 irAEs occurred, tislelizumab was permanently discontinued. Dose reductions were not allowed for tislelizumab. Bridging radiotherapy (BRT) was administered to patients based on the clinical characteristics including performance status, disease location and extension, and history of prior radiotherapy.

Response was assessed by the treating physician according to the Lugano criteria.^11^ The severity of cytokine release syndrome (CRS) and neurotoxicity (NT) was determined using the criteria established by Lee et al.^12^ Management of CRS and NT was adapted from the CARTOX working group.^13^

### 2.2 DNA and RNA sequencing

Targeted DNA sequencing was performed on formalin‐fixed paraffin‐embedded (FFPE) tumour samples, covering the genes involved in the 38‐gene algorithm, as previously described.^14^ RNA sequencing was conducted on qualified frozen tumour samples using Illumina Novaseq paired‐end sequencing (2 × 150 bp). Details for DNA and RNA sequencing are described in Supplementary Methods.

### 2.3 Tumour microenvironment analysis

TME analyses were performed at the pre‐CAR T‐cell infusion timepoint, referring to samples collected before leukapheresis and prior to zanubrutinib administration. Gene expression signatures of variable cells within the TME was based on the average expression of selected genes: Tnaive (*CD3D*, *CD3E*, *CD3G*, *CCR7*, *SELL*, *TCF7*, *LEF1*), Tcyto (*CD3D*, *CD3E*, *CD3G*, *CD8A*, *CD8B*, *CST7*, *PRF1*, *GZMA*, *GZMB*, *IFNG*, *CCL4*, *CCL3*), Tex (*CD3D*, *CD3E*, *CD3G*, *CD8A*, *CD8B*, *LAG3*, *HAVCR2*, *CTLA4*, *PDCD1*), Th17 (*CD3D*, *CD3E*, *CD3G*, *CD4*, *KLRB1*), Tfh (*CD3D*, *CD3E*, *CD3G*, *CD4*, *CXCL13*), Treg (*CD3D*, *CD3E*, *CD3G*, *CD4*, *IL2RA*, *FOXP3*), M1 *(S100A9*, *S100A8*, *IL1B, CXCL8*, *CCL3L1*), M2 (*C1QA*, *C1QB*, *C1QC*, *SLC40A1*, *APOC1*, *APOE*, *MMP9*, *GPNMB*, *CHI3L1*), cDC (*CPVL*, *RGCC*, *CCND1*, *CLEC9A*, *C1orf54*, *LAMP3*, *CCL19*, *CCL17*, *CCR7*, *BIR3*), pDC (*ITM2C*, *GZMB*, *JCHAIN*, *LILA4*, *IRF7*, *CLEC4C*), NK (*KLRC1*, *KLRD1*, *KLRF1*), CAF (*COL1A1*, *COL3A1*), Endo *(MCAM*, *CDH5*, *VWF*), MDSC (*CCR2*, *CXCR2*, *C5ar1*, *IL1B*, *CSF3R*, *IFITM1*, *ARG2*, *WFDC17*, *CD84*), progenitor Tex (*TCF7*, *BTLA*, *TNFRSF4*, *EEF1A1*, *SELL*, *CCR7*, *IL6R*, *IGFBP4*, *IGFL2*), intermediate Tex (*EOMES*, *CCR5*, *GZMA*, *GZMK*, *HLA‐DRB1*, *IFNG*), and terminal Tex (*TOX2*, *CXCR6*, *FASLG*, *GZMB*, *IL2RB*).^15^, ^16^

### 2.4 Gene set enrichment analysis

Gene sets from Gene Ontology (GO) database or Kyoto Encyclopedia of Genes and Genomes (KEGG) database were utilised for gene set enrichment analysis (GSEA).^17^ The analysis was carried out using the R package ‘clusterProfiler’ (version 4.2.2). Significance for terms or pathways was determined when the *p* value was < .05 with a false discovery rate < .25.

### 2.5 Immunohistochemistry

Immunostaining was performed on 5 µm sections of FFPE tumour samples using primary antibodies against CD3 (Abcam, ab16669, 1:100), CD8 (Abcam, ab237709, 1:2000), PD‐1 (Abcam, ab137132, 1:500), TOX2 (ThermoFisher, PA5‐40307, 1:100), CD163 (Abcam, ab182422,1:500), and TREM2 (Signalway Antibody, #29208, 1:200). Secondary antibodies included anti‐rabbit or anti‐mouse IgG (Dako, GV809, GV821). For cell counting, 5 randomly selected high‐power fields (HPFs) were analysed per section.

### 2.6 Statistical analysis

Continuous variables were reported as median (IQR) and categorical variables were described as *n* (%). Fisher's exact test was used to assess the association between categorical variables. *t*‐test was utilised to compare continuous variables across groups. Spearman's correlation test was applied to evaluate the association between two continuous variables. Progression‐free survival (PFS) was defined as the time from leukapheresis to first documented progression or death from any cause, and overall survival (OS) was measured from leukapheresis to death from any cause. Kaplan–Meier method was used to estimate PFS and OS rates, and log‐rank test was applied to assess the difference in PFS or OS between patient groups. Univariable and multivariable Cox regression models were constructed to evaluate associations with clinical outcomes. Variables with marginal associations (*p* < .1) with PFS or OS in univariable analysis were included in multivariable analysis. Results were considered statistically significant if *p* < .05. All statistical analyses were performed using R software (v4.1.2).

## 3 RESULTS

### 3.1 Baseline characteristics

Fifty‐four patients who received response‐adapted zanubrutinib plus tislelizumab upon CAR T‐cell therapy were included (Table S1). At leukapheresis, median age was 57 years (IQR 43–67). Twenty‐one patients (39%) were over 60 years of age, 29 (54%) were male, 22 (41%) had poor performance status, 38 (70%) had advanced Ann Arbor stage, 30 (56%) had multiple extranodal involvement, and 43 (80%) had elevated serum lactate dehydrogenase (LDH). Forty patients (74%) were diagnosed as diffuse large B‐cell lymphoma, including 8 with double‐ or triple‐hit lymphoma. Three patients (6%) were diagnosed as primary mediastinal large B‐cell lymphoma, one patient (2%) was diagnosed as primary central nervous system lymphoma, and 10 patients (18%) were diagnosed as transformed low‐grade lymphoma. Non‐germinal centre B‐cell subtype and BCL2/MYC double expressor immunophenotype were shown in 30 patients (56%) and 24 patients (44%), respectively. *TP53* mutations occurred in 21 patients (49%) of the 43 patients with available data. Seventeen patients (31%) underwent more than two prior lines of therapy before leukapheresis. Four patients (7%) received previous autologous stem‐cell transplantation (ASCT). Primary refractory disease occurred in 39 patients (72%). Tumour mass (single lymph nodes or conglomerates) with a maximal diameter (Dmax) greater than 4.0 cm was observed in 26 patients (48%) and Dmax greater than 7.5 cm was observed in 5 patients (9%) at leukapheresis. The time from leukapheresis to CAR T‐cell infusion in our study cohort was a median of 36 days (IQR 31–41).

### 3.2 Efficacy and safety

The median follow‐up time of the 54 patients was 23.6 months (range, 3.0–36.7 months). Upon evaluation on day 28, 36 patients who achieved CR continued with zanubrutinib monotherapy, 15 patients who attained PR proceeded with the combination of zanubrutinib and tislelizumab, and the 3 patients with stable disease (SD) or progressive disease (PD) received subsequent anti‐cancer treatment (Figure 1B). The overall response (OR) rates at day 28, month 3, and month 6 were 94% (CR 66%, PR 28%), 87% (CR 80%, PR 7%), and 80% (CR 76%, PR 4%), respectively (Figure 1C). Among the 15 patients who achieved a PR at day 28 post‐CAR‐T therapy, 10 patients subsequently converted to CR following the addition of tislelizumab. The estimated 2‐year PFS and OS rates were 68% (95% CI, 54–79) and 76% (95% CI, 62–85), respectively. Median PFS and median OS were not reached (Figure 1D).

AEs were summarised in Table S2. During treatment, no new safety signals were observed. The most common AEs of any grade were pyrexia (87%), neutropenia (87%), and thrombocytopenia (63%), with the most common high‐grade AEs as neutropenia (57%), anaemia (35%), and thrombocytopenia (26%), which were manageable and led to no discontinuation of treatment. CRS of any grade was present in 85% of patients, with grade ≥ 3 CRS occurring in 9%. Any grade of NT was present in 13% of the patients, and no patient presented with grade ≥ 3 NT. No discontinuation of treatment was observed in the 36 patients who received zanubrutinib monotherapy. Among the 15 patients who received the combination of zanubrutinib and tislelizumab, treatment discontinuation occurred in 5 patients. Specifically, 4 patients discontinued the treatment due to disease progression and 1 patient discontinued the treatment due to a severe skin rash.

We compared the survival and toxicity of the patients who received zanubrutinib alone to those who received in combination with tislelizumab, and no statistical difference was observed in PFS and OS between the two groups (Figure S1A). Combination therapy with tislelizumab did not result in an increase in AEs (Table S2).

### 3.3 Predictors of treatment outcome

To identify factors influencing treatment outcomes, we investigated associations between disease‐related features before CAR T‐cell infusion and outcome of response‐adapted zanubrutinib and tislelizumab. Factors related to unfavourable PFS and OS in patients treated with response‐adapted zanubrutinib and tislelizumab were identified by univariable (Table S3) and multivariable (Table S4) analyses. In this study, bulky disease was defined as having a tumour Dmax greater than 4.0 cm. The threshold was determined via the receiver operating characteristic (ROC) curve by optimising Youden's index (AUC = 0.7250, *p* = .0084; Figure S2A and B). Bulky disease (Dmax > 4.0 cm) was significantly associated with worse PFS (HR, 6.78; 95% CI, 1.94 to 23.71; *p* = .0005) and OS (HR, 4.34; 95% CI, 1.19 to 15.81; *p* = .0141) in a univariable Cox regression model. It remained a significant independent predictor of worse PFS (HR, 6.21; 95% CI, 1.76 to 21.89; *p* = .0045) and OS (HR, 3.77; 95% CI, 1.03 to 13.82; *p* = .0451) in a multivariable model adjusting for IPI score at leukapheresis. Of note, bridging radiotherapy (BRT) was administered to 44 patients (81%), with their characteristics detailed in Table S5. BRT was unrelated to PFS and OS in univariable or multivariable models.

Targeted sequencing was further performed in 43 patients of ZATI group. As identified by the LymphPlex,^14^ 21 patients (49%) were classified as *TP53* ^Mut^, 6 patients (14%) as MCD‐like, 4 patients (9%) as BN2‐like, 1 patient (2%) as N1‐like, 3 patients (7%) as EZB‐like, 2 patients (5%) as ST2‐like, and 6 patients (14%) as NOS. No significant differences in the CR rate, OR rate, PFS or OS was noted within distinct genetic subtypes (Figure S3A–D).

Using the gene expression data recently reported in untreated LBCL,^18^ we estimated the microenvironmental contexture for the LBCL of bulky disease (Dmax > 4.0 cm). Gene expression signatures for Tex cells, Treg cells, M2 macrophages, and MDSCs were analysed to characterise the tumour immune landscape. Patients with Dmax > 4.0 cm exhibited significantly higher expression of the Tex cell signature (*p* = .0329) and M2 macrophages (*p* = .0149) compared to those with non‐bulky disease (Figure S4), suggesting a more immunosuppressive microenvironment.

### 3.4 Impact on tumour microenvironment

**FIGURE 2:**
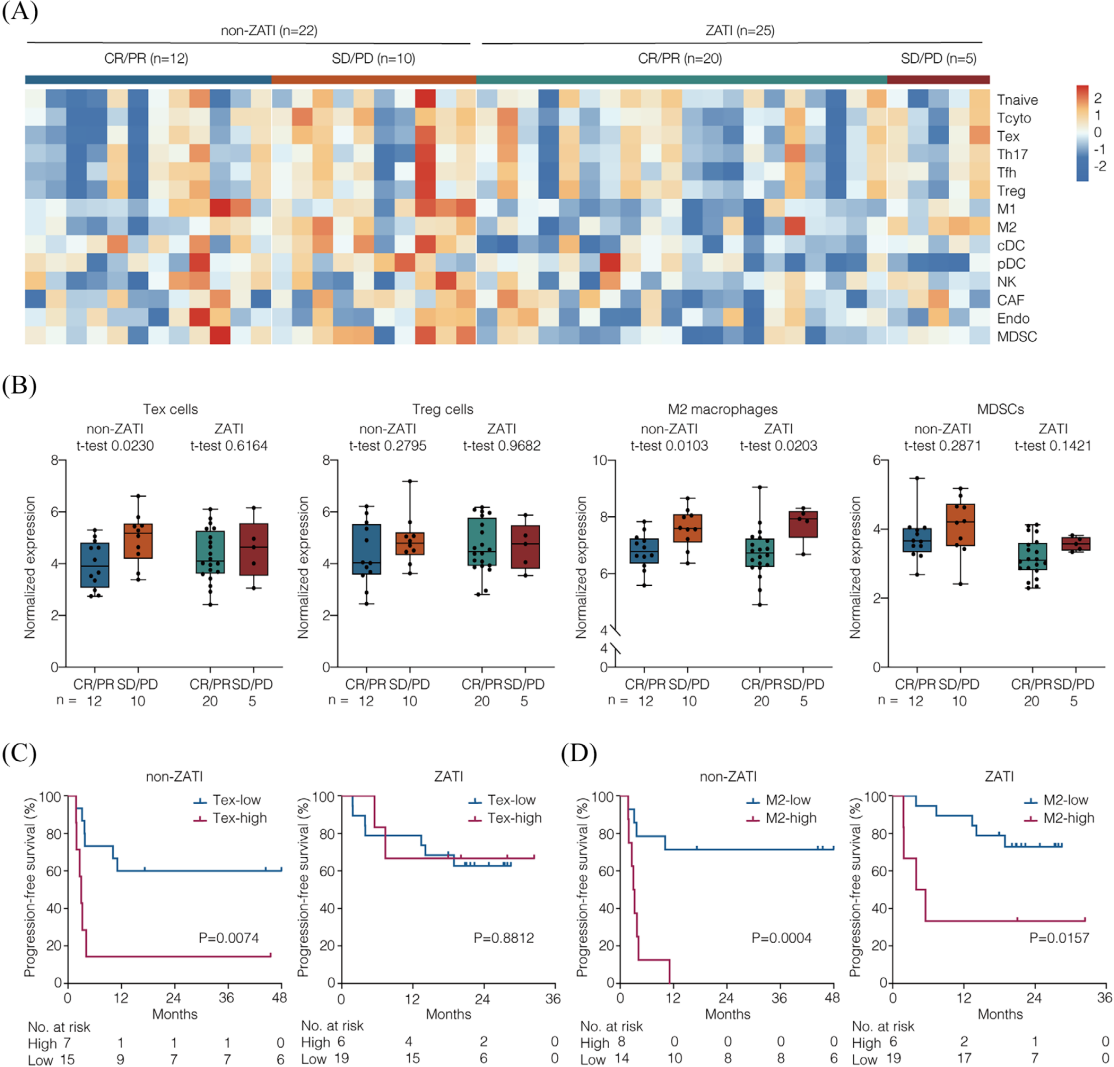
Pre‐treatment tumour microenvironment associated with clinical outcome. (A) Heat map of gene expression signatures measured by transcriptomic sequencing on available tumour biopsy specimens from patients in non‐ZATI group (*n* = 22; 12 patients with CR/PR; 10 patients with SD/PD) and ZATI group (*n* = 25; 20 patients with CR/PR; 5 patients with SD/PD). (B) Gene expression signatures of Tex cells, Treg cells, M2 macrophages, and MDSCs. The *p* values were calculated using the *t*‐test (2‐sided without adjustment). (C) PFS of patients receiving CAR T‐cell therapy, categorised according to the expression of Tex cells. (D) PFS of patients receiving CAR T‐cell therapy, categorised according to the expression of M2 macrophages. The *p* values were calculated using the log‐rank test.

To investigate the potential mechanisms underlying the effect of response‐adapted zanubrutinib and tislelizumab treatment on CAR T‐cell therapy, we analysed pre‐treatment TME contexture that may distinguish responders from non‐responders (Figure 2A). Responders were identified as patients achieving CR or PR, while non‐responders were those with SD or PD at the 3‐month post‐infusion evaluation. RNA sequencing was applied to tumour samples from 25 patients who received response‐adapted zanubrutinib and tislelizumab upon CAR T‐cell therapy (ZATI group). We compared the TME between patients who received zanubrutinib alone and those who received in combination with tislelizumab. No significant differences were observed in the immunosuppressive composition of Tex cells, Treg cells, M2 macrophages, or MDSCs between the two groups (Figure S1B). To better understand the effect of zanubrutinib plus tislelizumab, we used RNA sequencing data from 22 patients enrolled in clinical trials (NCT03355859, ChiCTR1800019661, NCT04812691, and ChiCTR2100047990) who did not receive combination therapy or underwent only a chemotherapy‐bridging regimen before CAR T‐cell infusion. These patients were designated as the non‐ZATI group for TME analyses. The non‐ZATI group comprised patients aged 18 years or older with histologically confirmed CD19+ R/R LBCL who had received prior systemic treatment, including rituximab and anthracycline‐based chemotherapy. All patients in this cohort underwent CAR T‐cell therapy with either axi‐cel or relma‐cel at our centre, without exposure to zanubrutinib or tislelizumab. Baseline characteristics of the non‐ZATI group were described in Table S6. Among these patients, six underwent a chemotherapy‐bridging regimen before CAR T‐cell therapy: four received the ICE regimen (etoposide, ifosfamide, and carboplatin), one received a combination of decitabine and G‐GemOx (obinutuzumab, gemcitabine, and oxaliplatin), and one received a combination of high‐dose methotrexate and temozolomide for a central nervous system relapse.

Gene expression signatures of the variable components was characterised by RNA sequencing data. The immunosuppressive subsets were assessed as previously reported,^3^, ^4^ including Tex cells, Treg cells, M2 macrophages, and MDSCs. In non‐ZATI group, the non‐responders presented increased expression of Tex cell signature, as compared to the responders (*p* = .0230; Figure 2B). Accordingly, inferior PFS was observed in the patients with higher Tex cell signature (*p* = .0074; Figure 2C). Of note, in ZATI group, neither response nor PFS was associated with the expression of Tex cell signature. With respect to M2 macrophage signature, the non‐responders presented increased expression of M2 macrophage signature in both non‐ZATI (*p* = .0103) and ZATI (*p* = .0203) groups. Consistently, inferior PFS was observed in the patients with higher M2 macrophage signature in both non‐ZATI (*p* = .0004) and ZATI (*p* = .0157) groups (Figure 2D). No association was observed in Treg cell signature or MDSC signature in terms of efficacy in either group. These results suggested that the regimen of response‐adapted zanubrutinib plus tislelizumab was effective against T‐cell exhaustion, but failed to overcome the immunosuppressive effects of M2 macrophage enrichment within the TME.

### 3.5 Effect on tumour‐infiltrating T cells

**FIGURE 3:**
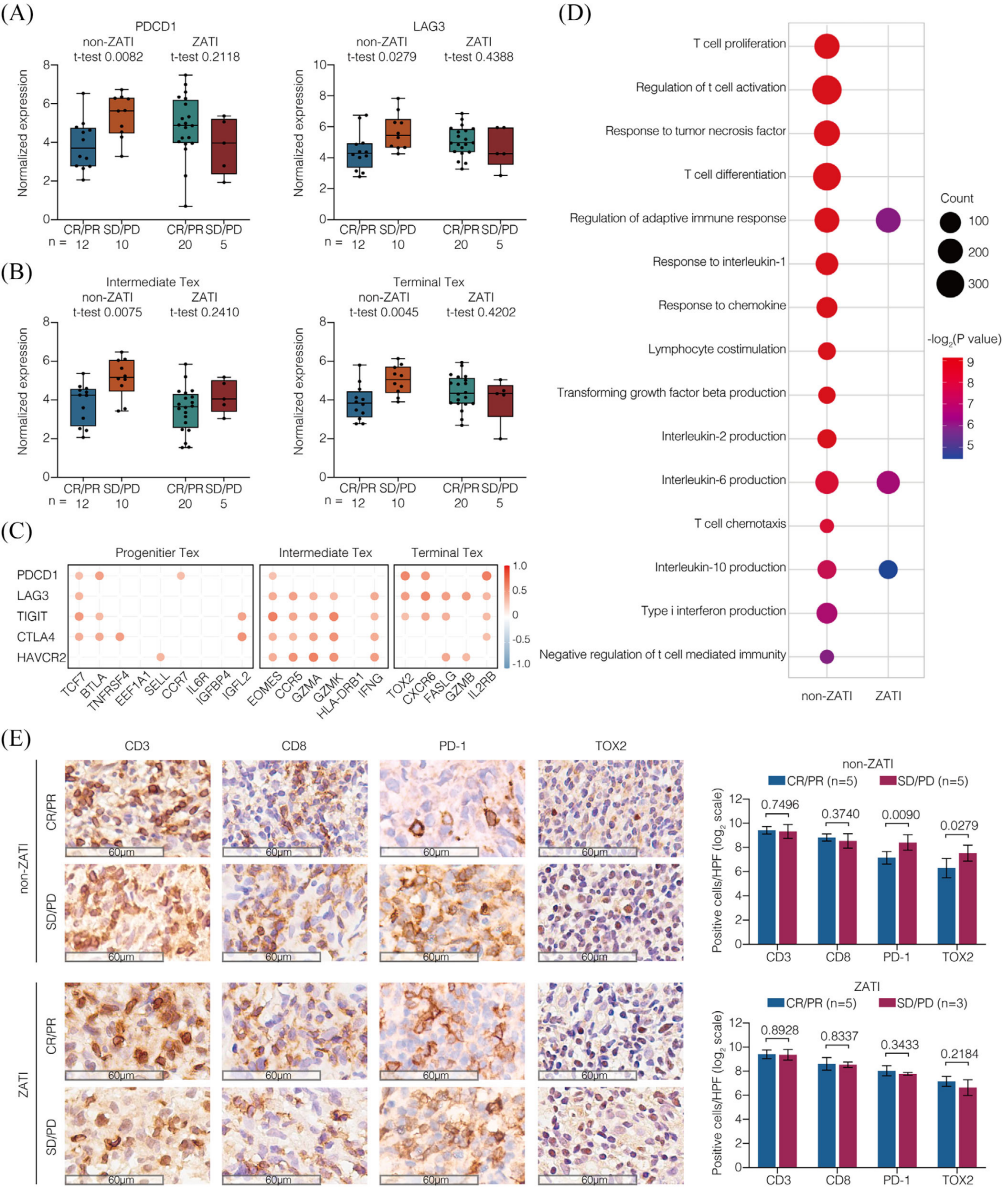
Exhausted tumour‐infiltrating T cells associated with response to CAR T‐cell therapy. (A) Expression of the exhaustion markers on T cells. (B) Gene expression signatures of Tex cells according to the developmental hierarchy. The *p* values were calculated using the *t*‐test (2‐sided without adjustment). (C) Correlation of exhaustion markers and gene expression signatures of Tex cells according to the developmental hierarchy. (D) GO terms enriched in the non‐responders. (E) IHC staining of CD3, CD8, PD‐1, and TOX2 expression on the tumour samples collected from the patients before treatment. The cells were counted from 5 randomly selected visions and subjected for statistical analysis. The *p* values were calculated using the *t*‐test (2‐sided without adjustment).

To further characterise heterogeneous Tex cells in CAR T‐cell therapy, Tex subsets were examined with the expression of specific genes, including progenitor Tex, intermediate Tex, and terminal Tex (Figure S5). In non‐ZATI group, non‐responders showed increased expression of inhibitory receptors *PDCD1* and *LAG3* compared to responders (*p* = .0082 and *p* = .0279, respectively; Figure 3A). As for Tex subsets, the signatures of intermediate Tex and terminal Tex were higher in the non‐responders than in responders (*p* = .0075 and *p* = .0045, respectively; Figure 3B). However, in ZATI group, neither the inhibitory receptors nor the Tex subsets was related to the response of CAR T‐cell therapy. These inhibitory receptors were predominantly expressed on intermediate and terminal Tex, with *PDCD1* strongly correlated with terminal Tex cells (Figure 3C). Furthermore, GSEA analysis was performed to distinguish altered biological functions of non‐responders from those of responders (Figure 3D). In non‐ZATI group, non‐responders presented dysregulation of anti‐tumour immunity including T‐cell functions (T‐cell proliferation, T‐cell activation, T‐cell differentiation, T‐cell chemotaxis, and lymphocyte co‐stimulation), cytokine signalling (response to tumour necrosis factor, response to interleukin‐1, response to chemokine, transforming growth factor beta production, interleukin‐2 production, interleukin‐6 production, interleukin‐10 production, and type I interferon production), and adaptive immune response (T‐cell mediated immunity). Remarkably, the T‐cell dysregulation linked to CAR T‐cell failure was largely overcome in ZATI group.

To validate these findings, we performed immunohistochemistry using tumour biopsy specimens before CAR T‐cell infusion (Figure 3E). In non‐ZATI group, non‐responders presented increased PD‐1^+^ and TOX2^+^ cells, as compared to the responders, indicating increased terminal Tex within the TME (*p* = .0090 and *p* = .0279, respectively). However, in ZATI group, neither terminal Tex signature was associated with response to CAR T‐cell therapy.

### 3.6 Effect on M2 macrophages with lipid metabolism alterations

**FIGURE 4:**
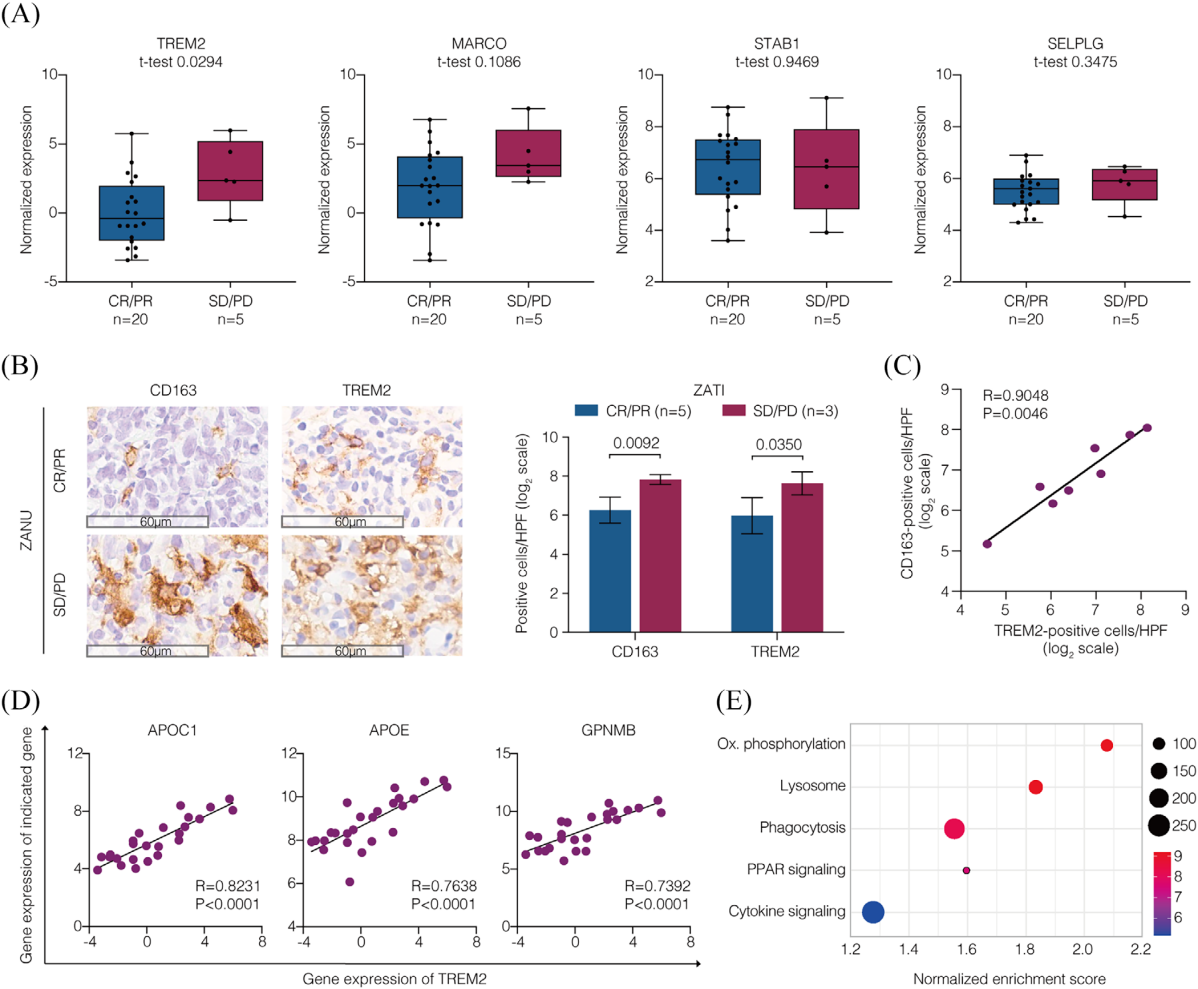
Influence of tumour‐infiltrating M2 macrophages with lipid‐associated alterations on CAR T‐cell therapy. (A) Expression of the negative regulators on M2 macrophages. (B) IHC staining of CD163 and TREM2 expression on the tumour samples collected from the patients before treatment. The cells were counted from 5 randomly selected visions and subjected for statistical analysis. The *p* values were calculated using the *t*‐test (2‐sided without adjustment). (C) Correlation of cell counts of Trem2^+^ cells and CD163^+^ cells by IHC. (D) Correlation of the expression of Trem2 and lipid‐metabolism‐related genes. Statistical significance of the Spearman's coefficient level (two‐sided *p* value) was calculated. (E) KEGG pathways enriched in the non‐responders.

To clarify the characteristics of the M2 macrophage enrichment within the TME that was not overcome by response‐adapted zanubrutinib plus tislelizumab treatment, we further featured the M2 macrophages with its negative regulators and distinct metabolic profile.^19^ In ZATI group, an increased expression of *TREM2* was associated with non‐responders (*p* = .0294), whereas no association was found between the expression of *MARCO*, *STAB1*, and *SELPLG* and response to CAR T‐cell therapy (Figure 4A). Immunohistochemistry on tumour biopsy specimens before CAR T‐cell infusion revealed elevated levels of CD163^+^ and TREM2^+^ cells in non‐responders compared to responders (*p* = .0092 and *p* = .0350, respectively; Figure 4B). Furthermore, TREM2^+^ cells showed strong positive correlation with CD163^+^ cells, suggesting an enrichment of TREM2^+^ M2 macrophages within the TME (Figure 4C). With respect to the metabolic profile, the expression of lipid metabolism‐associated genes *APOC1*, *APOE*, and *GPNMB* was highly correlated with *TREM2* expression (Figure 4D). The enriched gene signature was indicative of an active pathway initiated by phagocytosis, coupled with lipid metabolism (PPAR signalling and oxidative phosphorylation) and immunomodulation (cytokine signalling) in non‐responders (Figures 4E and S6). These results suggested that TREM2^+^ M2 macrophages characterised by lipid‐associated alterations were enriched in the TME, which may lead to resistance to CAR T‐cell therapy.

## 4 DISCUSSION

To our knowledge, this is the largest cohort to evaluate the chemotherapy‐free regimen zanubrutinib plus tislelizumab upon CAR T‐cell therapy in R/R LBCL. Importantly, this approach was designed to apply zanubrutinib and tislelizumab in a response‐adapted manner. This regimen not only proved to be effective and safe, but also functioned independently of genetic subtypes, including nearly 50% of high‐risk TP53^Mut^ subtype patients, rendering it more applicable for clinical practice.

The response‐adapted regimen of zanubrutinib and tislelizumab demonstrated promising efficacy and acceptable toxicity in enhancing the outcomes of CD19 CAR T‐cell therapy for patients with LBCL. Our study revealed a 2‐year PFS rate of 68% with this combination therapy, which is notably higher than the 39% rate reported in the ZUMA‐1 study using axi‐cel monotherapy in a similar patient population.^2^ The superior PFS observed in our study highlights the potential of zanubrutinib and tislelizumab to improve the outcomes of CAR T‐cell therapy. Seven patients in our cohort received CAR‐T therapy as a second‐line treatment, which might influence the observed response. Recent studies have explored the combination of CAR‐T therapy and BTKi in R/R DLBCL. A retrospective study of 21 high‐risk patients showed that zanubrutinib‐based bridging therapy prior to CAR‐T cell infusion achieved an ORR of 81% and a CR rate of 52.4%, with manageable toxicity.^10^ Another study reported that in six patients who received zanubrutinib one month post‐CAR‐T cell infusion, all PR cases converted to CR within six months, with a 100% sustained remission rate at 19.5 months.^9^ These findings suggested that BTKi may serve as both a bridging and maintenance strategy, but further clinical studies are needed to define the optimal timing and patient selection. Importantly, no new safety signals emerged during the treatment, and grade ≥ 3 CRS occurred in only 9% of patients, with no grade ≥ 3 NT reported. In our treatment approach, we implemented a bridging strategy of zanubrutinib with or without radiotherapy for all patients before CAR T‐cell infusion, aiming to minimise tumour burden. By effectively controlling the tumour mass, we may have attenuated the intensity of CRS and NT, contributing to the favourable safety profile observed in our cohort.

The immunosuppressive TME reduces the anti‐tumour effect of CAR T‐cells.^20^ BTKi can downregulate the expression of Tex markers, including PD‐1, and thereby enhance CAR T‐cell persistence and function.^4^, ^21^ Tex cells are categorised into progenitor, intermediate, and terminal stages according to the developmental hierarchy, with gradually increasing exhaustion markers and decreasing cytotoxic marker expression.^22^ TOX/TOX2 is a canonical transcription factor and is expressed on terminal Tex cells characterised by PD‐1 overexpression.^16^ Here we reported that zanubrutinib overcame TOX2^+^ terminal Tex cells during both immune‐bridging and immune‐maintenance therapy, correlating with reduced abundance of tumour‐infiltrating PD‐1‐overexpressing terminal Tex cells and good response to CAR T‐cell therapy. While zanubrutinib exhibits higher selectivity for BTK and has limited activity against interleukin‐2‐inducible T‐cell kinase (ITK), it may still provide benefits to CAR‐T cells. Additionally, inhibition of Zanubrutinib on other Tec‐family kinases could potentially modulate the T‐cell activation response.^23^ Furthermore, zanubrutinib and tislelizumab were administered as a combined regimen to patients who achieved PR at day 28. This approach significantly improved the outcome of CAR T‐cell therapy while minimising immunotherapy‐related toxicity. Therefore, negative impact of PD‐1^+^ and TOX2^+^ Tex cells were overcome, indicating that the response‐adapted zanubrutinib plus tislelizumab effectively abrogated T‐cell exhaustion in R/R LBCL upon CAR T‐cell therapy.

M2 macrophages, characterised by their immunosuppressive properties, may inhibit T‐cell functions and pose a barrier to effective CAR T‐cell therapy.^24^ In this study, we identified a subset of M2 macrophages with increased expression of cell surface receptor TREM2 that was associated with poor response to zanubrutinib plus tislelizumab.^19^ Interestingly, we found that bulky disease (Dmax > 4.0 cm) was an independent predictor for unfavourable outcome of CAR T‐cell therapy, linking to immunosuppressive TME alterations, mainly as enrichment of Tex cells and M2 macrophages, which explained why these patients were not responded to this regimen. Accumulating evidence indicates that TREM2^+^ macrophages drive immunosuppression, reduce response to ICIs, and cause poor prognosis in a variety of cancers, including lymphoma.^25^ The combination of anti‐TREM2 treatment with immunotherapy promotes expansion of immunostimulatory myeloid subsets, enhancing the anti‐tumour activity of immunotherapy such as ICIs or CAR T‐cell therapy.^26^ Furthermore, TREM2 expression was positively correlated with lipid‐associated genes *APOC1*, *APOE*, and *GPNMB*, coupled with active pathway of phagocytosis, PPAR signalling, and oxidative phosphorylation, suggesting a potential role of lipid metabolism by TREM2^+^ M2 macrophages.^27^ Since metabolic reprogramming affects immune cell function, how to utilise lipid metabolism‐associated agents to sustain CAR T‐cell efficacy needs further investigation.

This study has limitations. As a retrospective study based on real‐world clinical practice, treatment decisions were adapted according to individual patient responses and physician discretion, potentially introducing variability. Additionally, the relatively small sample size and single‐centre design could limit the generalisability of our findings. A prospective study involving response‐adapted zanubrutinib plus tislelizumab in CAR T‐cell therapy is ongoing (NCT05871684).

## 5 CONCLUSION

Response‐adapted zanubrutinib plus tislelizumab potentially enhances the therapeutic outcomes of CAR T‐cell therapy in patients with R/R LBCL with a favourable safety profile, through counteracting T‐cell exhaustion. Targeting TREM2^+^ M2 macrophages by reprogramming lipid metabolism could be an alternative approach to attenuate the immunosuppressive TME of R/R LBCL, providing a rationale for remodelling immunometabolism to optimise CAR T‐cell therapy.

## AUTHOR CONTRIBUTIONS

Rong Shen, Wei‐Guo Cao, Li Wang, Ling‐Shuang Sheng, Zi‐Xun Yan, and Wei‐Li Zhao contributed to conception and design; Rong Shen, Wei‐Guo Cao, Li Wang, Ling‐Shuang Sheng, Wen Wu, Peng‐Peng Xu, Shu Cheng, Xu‐Feng Jiang, Qi Song, Hong‐Mei Yi, Lei Li, Shu Cheng, Zi‐Xun Yan, and Wei‐Li Zhao provided study material or patients; all authors participated in the collection and assembly of data; Rong Shen, Wei‐Guo Cao, Li Wang, Ling‐Shuang Sheng, Yi‐Lun Zhang, Meng‐Ke Liu, Yan Dong, Yue Wang, Xiang‐Qin Weng, Zi‐Xun Yan, and Wei‐Li Zhao analysed and interpreted the data; Rong Shen and Wei‐Li Zhao wrote the manuscript; and all authors gave final approval of the manuscript and agreed to be accountable for all aspects of the work.

## CONFLICT OF INTEREST STATEMENT

All authors declare no competing interests.

### ETHICS STATEMENT

The study was approved by the Institutional Review Board of Ruijin Hospital, and informed consent was obtained in accordance with the Declaration of Helsinki.

## Supporting information



Supporting Information

## Data Availability

The data used in this study are available from the corresponding author on reasonable request.
