# Supplementary material for: Response‐adapted zanubrutinib and tislelizumab as a potential strategy to enhance CD19 CAR T‐cell therapy in relapsed/refractory large B‐cell lymphoma: A retrospective observational study
Source: Clin Transl Med. 2025 Apr 23;15(4):e70310. doi: 10.1002/ctm2.70310 (PMC12017895; doi:10.1002/ctm2.70310)
Supplement: Supplementary file 1 — Supporting Information [file CTM2-15-e70310-s001.docx]

Supplementary Materials for

Response-adapted zanubrutinib and tislelizumab as a potential strategy to enhance CD19 CAR T-cell therapy in relapsed/refractory large B-cell lymphoma: A retrospective observational study

Rong Shen^1*^, Wei-Guo Cao^2*^, Li Wang^1,3*^, Ling-Shuang Sheng^1*^, Yi-Lun Zhang^1^, Wen Wu^1^, Peng-Peng Xu^1^, Shu Cheng^1^, Meng-Ke Liu^1^, Yan Dong^1^, Yue Wang^1^, Xiang-Qin Weng^1^, Xu-Feng Jiang^4^, Qi Song^5^, Hong-Mei Yi^6^, Lei Li^7^, Sheng Chen^8^, Zi-Xun Yan^1#^, Wei-Li Zhao^1,3#^

**^#^** Correspondence to: Wei-Li Zhao, Email: zhao.weili@yahoo.com, Zi-Xun Yan, Email: yanzixun125@163.com.

**This PDF file includes:**

Supplementary Methods

Supplementary Tables (Table S1-S6)

Supplementary Figures (Figure S1-S6)

**Supplementary Methods**

**DNA sequencing**

Formalin-fixed paraffin-embedded (FFPE) tumor biopsy samples were obtained from all patients before CAR-T infusion for pathology review and targeted DNA sequencing. Genomic DNA was extracted from FFPE samples using a GeneRead DNA FFPE Tissue Kit (Qiagen, Hilden, Germany) according to the manufacturer’s guidelines.

Before sequencing, genomic DNA was cut into fragments with ~200 bp using a focused ultrasonicator (No. M220, Covaris, Woburn, MA, USA). DNA quantity was determined using a Nanodrop 8,000 UV-Vis spectrometer (NanoDrop Technologies, Wilmington, DE, USA), Qubit 2.0 Fluorometer (Life Technologies, Carlsbad, CA, USA), and 2200 TapeStation Instrument (Agilent Technologies, Santa Clara, CA, USA).

The paired-end reads of targeted sequencing were aligned to the Human Genome Reference Consortium build 38 (GRCh38) using Burrows-Wheeler Aligner (BWA, version 0.5.9-tpx). Raw sequencing data were processed by using Trimmomatic (version 3.6) software to remove sequencing adapters and low-quality reads referring to the joint sequence fragments of the 3’ end and low-quality fragments with Q value < 25 and fragments with < 35 bp. Samtools (v0.1.18) ^1^, picard (v1.93), and Genome Analysis Toolkit (GATK, v4.1.4.0) were used for BAM file handling, local realignment, base recalibration and calling variants, respectively. Mutations in the coding region were annotated using the Annovar software (version 2017-07-17) ^2^.

Variants with depth > 10 and VAF > 0.05 were first filtered with variants detected by pooled results of selected peripheral blood samples, as described in our previous study ^3^. Then The non-synonymous SNVs, indels and splicing sites were preserved with following criteria: (i) reported as somatic mutations in our previous studies ^4-6^; (ii) commonly considered as hotspot mutations like MYD88L265P; (iii) categorized into tier I and II variants according to Guideline for Evidence-Based Categorization of Somatic Variants ^7^; (iv) not observed in 1000 genome (1KG) project, observed in less than 0.01 in databases of 1KG project, or observed in less than 0.05 in databases of 1KG project associated with recurrently observation (n > 5) in hematological tumors in the COSMIC (v77) database. Variants were excluded with following criteria: (i) categorized into tier IV variants according to Guideline for Evidence-Based Categorization of Somatic Variants; (ii) observed in paired peripheral blood samples.

**RNA sequencing**

Fresh frozen samples were preserved for patients with residual tissue available from the biopsies before CAR-T infusion for RNA sequencing. Total RNA was extracted from fresh frozen samples using Trizol and a RNeasy Mini Kit (Qiagen, Hilden, Germany).

RNA quality was assessed by RNA 6000 NanoChip using 2100 Bioanalyzer (Agilent Technologies) and RNA concentration was measured using Qubit® RNA HS Assay Kit by Qubit® 2.0 Fluorometer (Life Technologies, Grand Island, NY, USA) according to the manufacturer’s instructions.

RNA purification, reverse transcription, library construction and sequencing were performed in WuXi NextCODE according to the manufacturer’s instructions (Illumina San Diego, CA, USA). PolyA mRNA was purified from total RNA using oligo-dT-attached magnetic beads and then fragmented by fragmentation buffer. The synthesized cDNA was subjected to end-repair, phosphorylation, and ‘A’ base addition according to Illumina’s library construction protocol. Then Illumina sequencing adapters were added to both size of the cDNA fragments. After PCR amplification for DNA enrichment, the target fragments of 200-300 bp were cleaned up. After library construction, Qubit (Thermo Fisher Scientific) was used to quantify concentration of the resulting sequencing libraries, while the size distribution was analyzed using Agilent BioAnalyzer 2100 (Agilent). After library validation, Illumina cBOT cluster generation system with HiSeq PE Cluster Kits (Illumina) was used to generate clusters. High-throughput RNA-sequencing was performed using Illumina Novaseq paired-end sequencing (2 × 150 bp) (Illumina, California, USA).

The paired-end reads were pseudo-aligned to GRCh38 transcriptome (Ensembl version 106) and quantified using Kallisto software (v0.46.0) ^8^. Voom function from R package “limma” (v3.38.3) was used to remove batch effect and normalize raw reads R package “clusterProfiler” (v3.10.1) was used for Gene Ontology analysis and GSEA analysis.

**References**

1. Danecek P, Bonfield JK, Liddle J, et al. Twelve years of SAMtools and BCFtools. *Gigascience*. 2021;10(2)

2. Wang K, Li M, Hakonarson H. ANNOVAR: functional annotation of genetic variants from high-throughput sequencing data. *Nucleic Acids Res*. 2010;38(16):e164.

3. Zhang MC, Tian S, Fu D, et al. Genetic subtype-guided immunochemotherapy in diffuse large B cell lymphoma: The randomized GUIDANCE-01 trial. *Cancer Cell*. 2023;41(10):1705-1716.e5.

4. Huang YH, Cai K, Xu PP, et al. CREBBP/EP300 mutations promoted tumor progression in diffuse large B-cell lymphoma through altering tumor-associated macrophage polarization via FBXW7-NOTCH-CCL2/CSF1 axis. *Signal Transduct Target Ther*. 2021;6(1):10.

5. Qin W, Fu D, Shi Q, et al. Molecular Heterogeneity in Localized Diffuse Large B-Cell Lymphoma. *Front Oncol*. 2021;11:638757.

6. Shen R, Fu D, Dong L, et al. Simplified algorithm for genetic subtyping in diffuse large B-cell lymphoma. *Signal Transduct Target Ther*. 2023;8(1):145.

7. Li MM, Datto M, Duncavage EJ, et al. Standards and Guidelines for the Interpretation and Reporting of Sequence Variants in Cancer: A Joint Consensus Recommendation of the Association for Molecular Pathology, American Society of Clinical Oncology, and College of American Pathologists. *J Mol Diagn*. 2017;19(1):4-23.

8. Bray NL, Pimentel H, Melsted P, Pachter L. Near-optimal probabilistic RNA-seq quantification. *Nat Biotechnol*. 2016;34(5):525-7.

**Supplementary Tables and Figures**

| **Supplementary Table S1. Baseline characteristics of patients treated with response-adapted zanubrutinib plus tislelizumab (ZATI group) in this study.** | |
| --- | --- |
| **Characteristic** | **No. of patients (%)** |
| Median age (IQR) | 57 (43-67) |
| Age-categorical, *n* (%) |  |
| ≤ 60 | 33 (61) |
| > 60 | 21 (39) |
| Sex, *n* (%) |  |
| Male | 29 (54) |
| Female | 25 (46) |
| ECOG PS, *n* (%) |  |
| 0-1 | 32 (59) |
| ≥ 2 | 22 (41) |
| Ann Arbor stage, *n* (%) |  |
| I or II | 16 (30) |
| III or IV | 38 (70) |
| Sites of extranodal involvement, *n* (%) |  |
| 0-1 | 24 (44) |
| ≥ 2 | 30 (56) |
| LDH > ULN, *n* (%) | 43 (80) |
| Disease type, *n* (%) |  |
| DLBCL | 40 (74) |
| PMBCL | 3 (6) |
| PCNSL | 1 (2) |
| Transformed low-grade lymphoma | 10 (18) |
| Cell of origin^a^, *n* (%) |  |
| GCB | 24 (44) |
| Non-GCB | 30 (56) |
| Double expressor, *n* (%) | 24 (44) |
| Double or triple-hit, *n* (%) | 8 (15) |
| *TP53* mutations^b^, *n* (%) | 21 (49) |
| Prior lines of therapy, *n* (%) |  |
| 1 | 7 (13) |
| 2 | 30 (56) |
| > 2 | 17 (31) |
| Previous ASCT, *n* (%) | 4 (7) |
| Primary refractory, *n* (%) | 39 (72) |
| Dmax > 4 cm, *n* (%) | 26 (48) |
| Dmax > 7.5 cm, *n* (%) | 5 (9) |
| Time from leukapheresis to CAR T-cell infusion (IQR) | 36 (31–41) days |
| *ASCT* autologous stem-cell transplantation, *DLBCL* diffuse large B-cell lymphoma, *Dmax* maximal diameter, *ECOG PS* Eastern Cooperative Oncology Group performance status, *GCB* germinal center B cell, *IQR* interquartile range, *LDH* lactate dehydrogenase, *PCNSL* primary central nervous system lymphoma, *PMBCL* primary mediastinal B-cell lymphoma, *ULN* upper limit of normal. | |
| ^a^ Hans algorithm. |  |
| ^b^ Percentage of those with data available. |  |

| **Supplementary Table S2. Adverse events in patients** **treated with response-adapted zanubrutinib plus tislelizumab upon CAR T-cell therapy.**   \| **Event** \| **Overall (n=54)** \| \|  \| **Zanu (n=36)^a^** \| \|  \| **Zanu+Tis (n=15)^b^** \| \| \| --- \| --- \| --- \| --- \| --- \| --- \| --- \| --- \| --- \| \| **Any** \| **Grade≥3** \|  \| **Any** \| **Grade≥3** \|  \| **Any** \| **Grade≥3** \| \| Pyrexia \| 47 (87) \| 11 (20) \|  \| 31 (86) \| 7 (19) \|  \| 13 (87) \| 3 (20) \| \| Neutropenia \| 47 (87) \| 31 (57) \|  \| 33 (92) \| 21 (58) \|  \| 11 (73) \| 8 (53) \| \| Thrombocytopenia \| 34 (63) \| 14 (26) \|  \| 22 (61) \| 9 (25) \|  \| 9 (60) \| 4 (27) \| \| Anemia \| 33 (61) \| 19 (35) \|  \| 22 (61) \| 12 (33) \|  \| 8 (53) \| 5 (33) \| \| Decreased appetite \| 32 (59) \| 0 (0) \|  \| 22 (61) \| 0 (0) \|  \| 8 (53) \| 0 (0) \| \| Hypotension \| 30 (56) \| 5 (9) \|  \| 19 (53) \| 2 (6) \|  \| 9 (60) \| 2 (13) \| \| Fatigue \| 30 (56) \| 0 (0) \|  \| 20 (56) \| 0 (0) \|  \| 7 (47) \| 0 (0) \| \| Hypokalema \| 30 (56) \| 0 (0) \|  \| 19 (53) \| 0 (0) \|  \| 9 (60) \| 0 (0) \| \| Nausea \| 29 (54) \| 0 (0) \|  \| 18 (50) \| 0 (0) \|  \| 9 (60) \| 0 (0) \| \| Hypoalbuminemia \| 25 (46) \| 1 (2) \|  \| 15 (42) \| 0 (0) \|  \| 8 (53) \| 0 (0) \| \| Hypocalcemia \| 20 (37) \| 0 (0) \|  \| 13 (36) \| 0 (0) \|  \| 6 (40) \| 0 (0) \| \| ALT or AST elevation \| 16 (30) \| 0 (0) \|  \| 10 (28) \| 0 (0) \|  \| 3 (20) \| 0 (0) \| \| Arrhythmia \| 15 (28) \| 0 (0) \|  \| 9 (25) \| 0 (0) \|  \| 5 (33) \| 0 (0) \| \| Skin rash \| 12 (22) \| 0 (0) \|  \| 8 (22) \| 0 (0) \|  \| 3 (20) \| 0 (0) \| \| Vomiting \| 9 (17) \| 0 (0) \|  \| 6 (17) \| 0 (0) \|  \| 2 (13) \| 0 (0) \| \| Hypothyroidism \| 8 (15) \| 0 (0) \|  \| 5 (14) \| 0 (0) \|  \| 3 (20) \| 0 (0) \| \| Headache \| 8 (15) \| 0 (0) \|  \| 5 (14) \| 0 (0) \|  \| 2 (13) \| 0 (0) \| \| Adverse event of special interest \|  \|  \|  \|  \|  \|  \|  \|  \| \| CRS \| 46 (85) \| 4 (7) \|  \| 30 (83) \| 2 (6) \|  \| 13 (87) \| 1 (7) \| \| NT \| 7 (13) \| 1 (2) \|  \| 4 (11) \| 0 (0) \|  \| 3 (20) \| 1 (7) \| |
| --- | --- | --- | --- | --- | --- | --- | --- | --- | --- | --- | --- | --- | --- | --- | --- | --- | --- | --- | --- | --- | --- | --- | --- | --- | --- | --- | --- | --- | --- | --- | --- | --- | --- | --- | --- | --- | --- | --- | --- | --- | --- | --- | --- | --- | --- | --- | --- | --- | --- | --- | --- | --- | --- | --- | --- | --- | --- | --- | --- | --- | --- | --- | --- | --- | --- | --- | --- | --- | --- | --- | --- | --- | --- | --- | --- | --- | --- | --- | --- | --- | --- | --- | --- | --- | --- | --- | --- | --- | --- | --- | --- | --- | --- | --- | --- | --- | --- | --- | --- | --- | --- | --- | --- | --- | --- | --- | --- | --- | --- | --- | --- | --- | --- | --- | --- | --- | --- | --- | --- | --- | --- | --- | --- | --- | --- | --- | --- | --- | --- | --- | --- | --- | --- | --- | --- | --- | --- | --- | --- | --- | --- | --- | --- | --- | --- | --- | --- | --- | --- | --- | --- | --- | --- | --- | --- | --- | --- | --- | --- | --- | --- | --- | --- | --- | --- | --- | --- | --- | --- | --- | --- | --- | --- | --- | --- | --- | --- | --- | --- | --- | --- | --- | --- | --- | --- | --- | --- | --- | --- | --- | --- | --- | --- | --- | --- | --- | --- |
| Abbreviations: ALT, alanine aminotransferase; AST, Aspartate Aminotransferase; CRS, cytokine release syndrome; NT, neurotoxicity; Zanu, zanubrutinib; Tis, tislelizumab. |
| ^a^Zanubrutinib monotherapy after the evaluation on day 28 |
| ^b^Combination of zanubrutinib and tislelizumab after the evaluation on day 28 |
| *Listed are adverse events that occurred in at least 15% of all patients, along with the events of cytokine release syndrome and neurotoxicity. |

**Supplementary Table S3. Characteristics associated with PFS and OS in patients treated with response-adapted zanubrutinib plus tislelizumab upon CAR T-cell therapy by univariable analysis.**

| **Characteristic** | **PFS** | |  | **OS** | |
| --- | --- | --- | --- | --- | --- |
|  | ***P*** | **HR (95% CI)** |  | ***P*** | **HR (95% CI)** |
| Sex |  |  |  |  |  |
| Male *v* female | 0.9706 | 0.98 (0.38-2.55) |  | 0.5226 | 1.43 (0.47-4.39) |
| IPI |  |  |  |  |  |
| 3-5 *v* 0-2 | 0.0990 | 2.42 (0.79-7.42) |  | 0.0332 | 4.13 (0.91-18.62) |
| Cell of origin |  |  |  |  |  |
| Non-GCB *v* GCB | 0.3906 | 1.53 (0.57-4.15) |  | 0.6410 | 1.30 (0.43-3.98) |
| Double expressor |  |  |  |  |  |
| Yes *v* no | 0.4383 | 1.46 (0.56-3.78) |  | 0.1923 | 2.08 (0.68-6.35) |
| Prior lines of therapy |  |  |  |  |  |
| > 2 *v* ≤ 2 | 0.6135 | 1.30 (0.48-3.51) |  | 0.5203 | 1.45 (0.47-4.44) |
| Primary refractory |  |  |  |  |  |
| Yes *v* no | 0.5730 | 1.37 (0.45-4.20) |  | 0.6635 | 1.32 (0.36-4.81) |
| Maximal diameter of tumor masse | |  |  |  |  |
| > 4 cm *v* ≤ 4cm | 0.0005 | 6.78 (1.94-23.71) |  | 0.0141 | 4.34 (1.19-15.81) |
| Bridging-radiotherapy | |  |  |  |  |
| Yes *v* no | 0.3482 | 1.92 (0.44-8.39) |  | 0.6947 | 1.34 (0.30-6.04) |

*GCB* germinal center B cell, *HR* hazard ratio, *PD* progressive disease, *PR* partial response, *SD* stable disease.

| **Supplementary Table S4. Characteristics associated with PFS and OS in multivariable models of patients treated with response-adapted zanubrutinib plus tislelizumab upon CAR-T cell therapy.** | | | | | |
| --- | --- | --- | --- | --- | --- |
| **Characteristic** | **PFS** | |  | **OS** | |
|  | ***P*** | **HR (95% CI)** |  | ***P*** | **HR (95% CI)** |
| IPI |  |  |  |  |  |
| 3-5 *v* 0-2 | 0.2727 | 1.88 (0.61-5.81) |  | 0.108 | 3.46 (0.76-15.73) |
| Dmax of tumor masse |  |  |  | |  |
| > 4 cm *v* ≤ 4cm | 0.0045 | 6.21 (1.76-21.89) |  | 0.0451 | 3.77 (1.03-13.82) |

*Dmax* maximal diameter, *HR* hazard ratio.

**Supplementary Table S5. Characteristics of patients according to the treatment of bridging radiotherapy.**

| **Characteristic** | **Received BRT**  **(n = 44)** | **No BRT**  **(n = 10)** | ***P*** |
| --- | --- | --- | --- |
| Age-categorical, *n* (%) |  |  | 0.7230 |
| ≤ 60 | 26 (59) | 7 (70) |  |
| > 60 | 18 (41) | 3 (30) |  |
| Sex, *n* (%) |  |  | 0.7363 |
| Male | 23 (52) | 6 (60) |  |
| Female | 21 (48) | 4 (40) |  |
| ECOG PS, *n* (%) |  |  | 1.0000 |
| 0-1 | 26 (59) | 6 (60) |  |
| ≥ 2 | 18 (41) | 4 (40) |  |
| Ann Arbor stage, *n* (%) |  |  | 1.0000 |
| I or II | 13 (30) | 3 (30) |  |
| III or IV | 31 (70) | 7 (70) |  |
| Sites of extranodal involvement, *n* (%) |  |  | 0.4832 |
| 0-1 | 21 (48) | 3 (30) |  |
| ≥ 2 | 23 (52) | 7 (70) |  |
| LDH > ULN, *n* (%) | 37 (84) | 6 (60) | 0.1853 |
| Disease type, *n* (%) |  |  | 0.8057 |
| DLBCL | 32 (73) | 8 (80) |  |
| PMBCL | 3 (7) | 0 (0) |  |
| PCNSL | 1 (2) | 0 (0) |  |
| Transformed low-grade lymphoma | 8 (18) | 2 (20) |  |
| Cell of origin^a^, *n* (%) |  |  | 0.7363 |
| GCB | 19 (43) | 5 (50) |  |
| Non-GCB | 25 (57) | 5 (50) |  |
| Double expressor, *n* (%) | 19 (43) | 5 (50) | 0.7363 |
| Double or triple-hit, *n* (%) | 6 (14) | 2 (20) | 0.6317 |
| *TP53* mutations^b^, *n* (%) | 18 (50) | 3 (43) | 1.0000 |
| Prior lines of therapy, *n* (%) |  |  | 0.7075 |
| ≤ 2 | 31 (70) | 6 (60) |  |
| > 2 | 13 (30) | 4 (40) |  |
| Previous ASCT, *n* (%) | 3 (7) | 1 (10) | 0.5707 |
| Primary refractory, *n* (%) | 33 (75) | 6 (60) | 0.4376 |
| Dmax > 4 cm, *n* (%) | 23 (52) | 3 (30) | 0.2975 |
| Dmax > 7.5 cm, *n* (%) | 5 (11) | 0 (0) | 0.5707 |

*ASCT* autologous stem-cell transplantation, *BRT* bridging radiotherapy, *DLBCL* diffuse large B-cell lymphoma, *Dmax* maximal diameter, *ECOG PS* Eastern Cooperative Oncology Group performance status, *GCB* germinal center B cell, *IQR* interquartile range, *LDH* lactate dehydrogenase, *PCNSL* primary central nervous system lymphoma, *PMBCL* primary mediastinal B-cell lymphoma, *ULN* upper limit of normal.

^a^ Hans algorithm.

^b^ Percentage of those with data available.

**Supplementary Table S6. Baseline characteristics of the non-ZATI group (n=22).**

| **Characteristic** | **No. of patients (%)** |
| --- | --- |
| Median age (IQR) | 57 (50-62) |
| Age-categorical, *n* (%) |  |
| ≤ 60 | 15 (68) |
| > 60 | 7 (32) |
| Sex, *n* (%) |  |
| Male | 14 (64) |
| Female | 8 (36) |
| ECOG PS, *n* (%) |  |
| 0-1 | 19 (86) |
| ≥ 2 | 3 (14) |
| Ann Arbor stage, *n* (%) |  |
| I or II | 2 (9) |
| III or IV | 20 (91) |
| Sites of extranodal involvement, *n* (%) |  |
| 0-1 | 8 (36) |
| ≥ 2 | 14 (64) |
| LDH > ULN, *n* (%) | 17 (77) |
| Disease type, *n* (%) |  |
| DLBCL | 20 (91) |
| PMBCL | 1 (4) |
| PCNSL | 0 (0) |
| Transformed low-grade lymphoma | 1 (4) |
| Cell of origin^a^, *n* (%) |  |
| GCB | 6 (27) |
| Non-GCB | 16 (73) |
| Double expressor, *n* (%) | 8 (36) |
| Double or triple-hit, *n* (%) | 1 (4) |
| *TP53* mutations^b^, *n* (%) | 9 (43) |
| Prior lines of therapy, *n* (%) |  |
| 1 | 7 (32) |
| 2 | 5 (23) |
| > 2 | 10 (45) |
| Previous ASCT, *n* (%) | 2 (9) |
| Primary refractory, *n* (%) | 14 (64) |
| Dmax > 4 cm, *n* (%) | 14 (64) |
| Dmax > 7.5 cm, *n* (%) | 3 (14) |
| Bridging therapy |  |
| No-bridging | 16 (73) |
| Chemotherapy-bridging | 6 (27) |
| Time from leukapheresis to CAR T-cell infusion (IQR) | 28 (26–31) days |
| *ASCT* autologous stem-cell transplantation, *DLBCL* diffuse large B-cell lymphoma, *Dmax* maximal diameter, *ECOG PS* Eastern Cooperative Oncology Group performance status, *GCB* germinal center B cell, *IQR* interquartile range, *LDH* lactate dehydrogenase, *PCNSL* primary central nervous system lymphoma, *PMBCL* primary mediastinal B-cell lymphoma, *ULN* upper limit of normal. | |
| ^a^ Hans algorithm. |  |
| ^b^ Percentage of those with data available. |  |

**Supplementary Figure S1. Comparison of the patients who received zanubrutinib alone or in combination with tislelizumab in those who achieved CR or PR at month 3.**


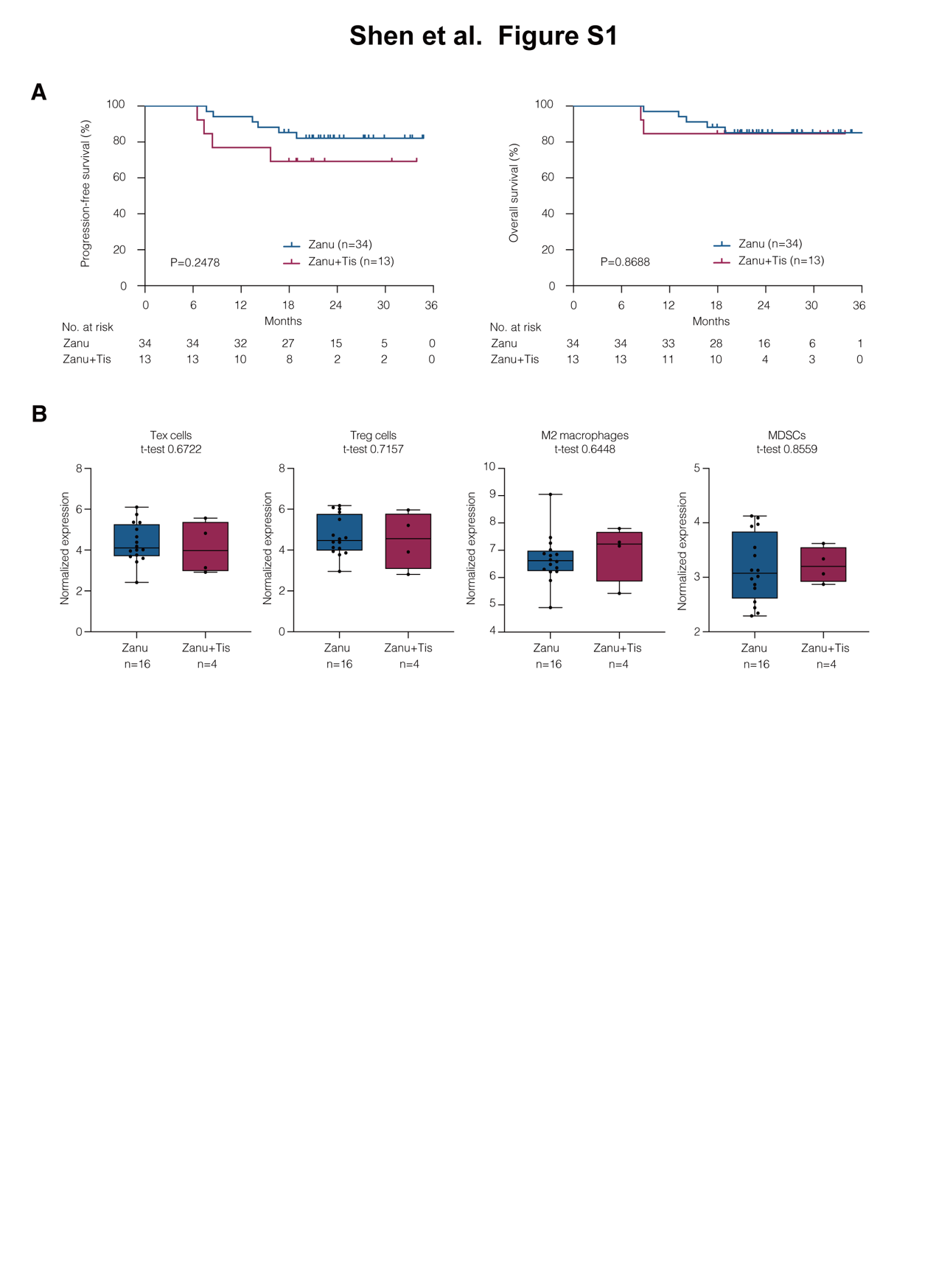


PFS and OS (A) within subgroups who received zanubrutinib alone (n = 34 ) or in combination with tislelizumab (n = 13) in those who achieved CR or PR at month 3 (n = 43). Comparison of gene expression signatures of Tex cells, Treg cells, M2 macrophages, and MDSCs, categorized according to subgroups who received zanubrutinib alone (n = 16 ) or in combination with tislelizumab (n = 4).

**Supplementary Figure S2. ROC curve analysis and survival analysis according to disease bulk.**

**
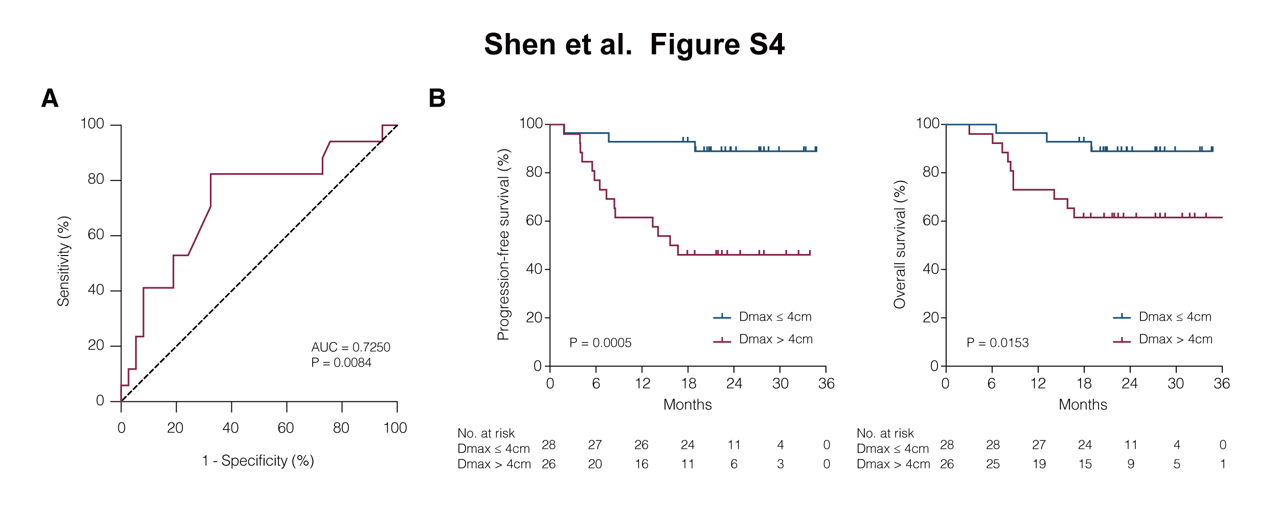
**

ROC curve analysis (A) and survival analysis on PFS and OS (B) according to disease bulk.

**Supplementary Figure S3. Response and survival within distinct genetic subtypes in patients treated with response-adapted zanubrutinib plus tislelizumab upon CAR T-cell therapy.**

**
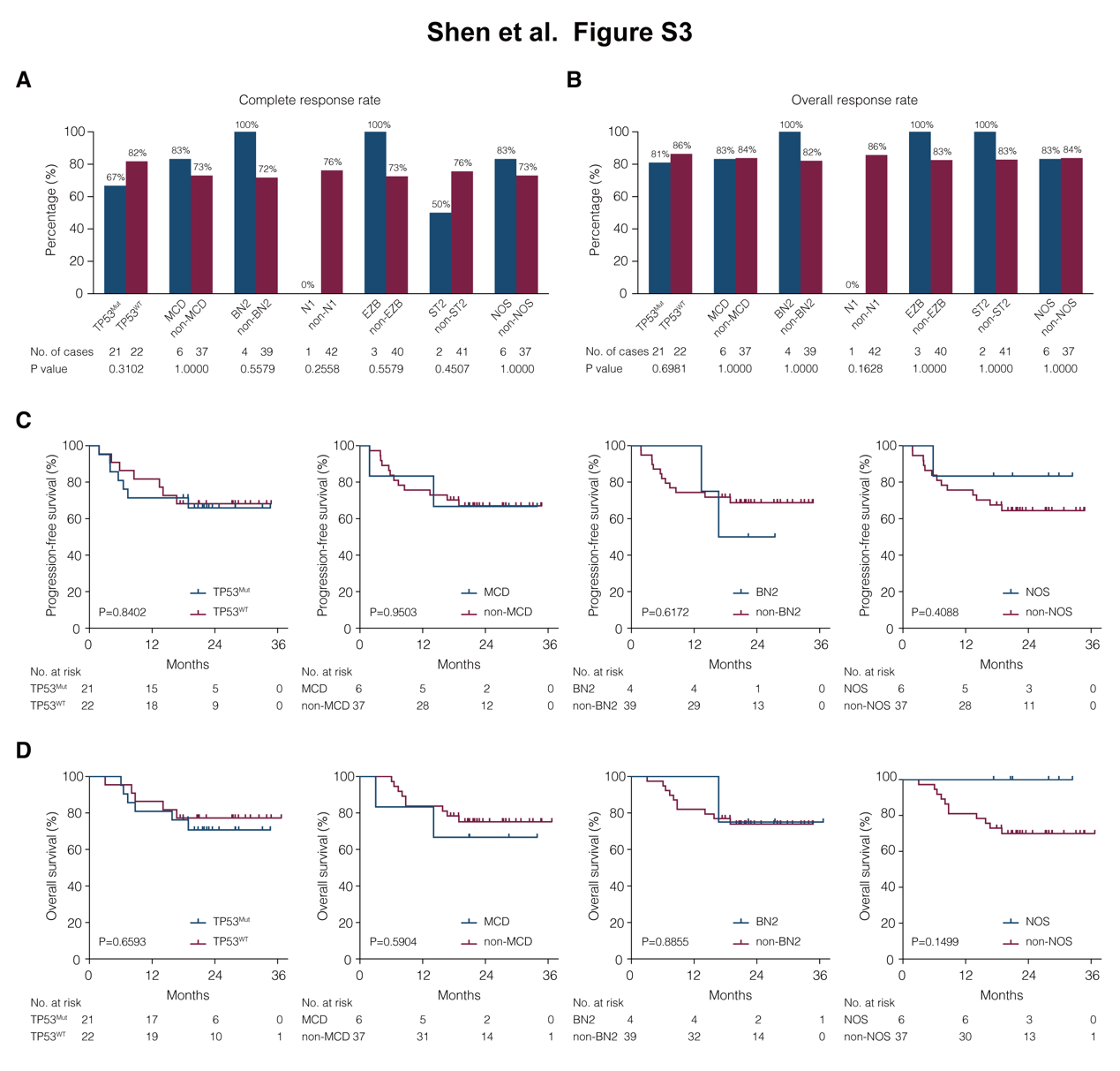
**

Comparison of CR rate (A), OR rate (B), PFS (C), and OS (D) within distinct genetic subtypes in patients treated with response-adapted zanubrutinib plus tislelizumab upon CAR T-cell therapy (n = 43). The subtypes with more than 3 cases were shown for PFS and OS.

**Supplementary Figure S4. Gene expression signatures for Tex cells, Treg cells, M2 macrophages, and MDSCs according to disease bulk.**


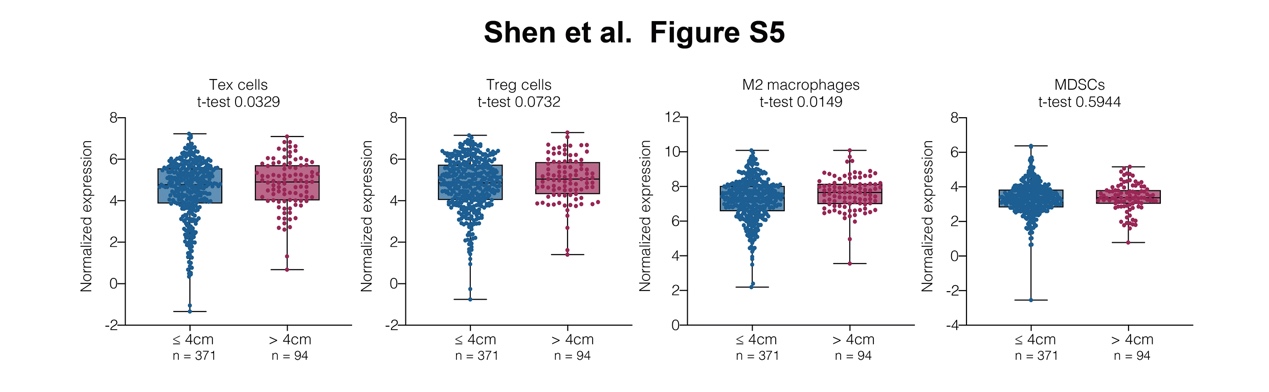


Comparison of gene expression signatures of Tex cells, Treg cells, M2 macrophages, and MDSCs, categorized according to disease bulk in untreated LBCL.

**Supplementary Figure S5. Heat map of gene expression for exhaustion markers and developmental stages of Tex cells.**


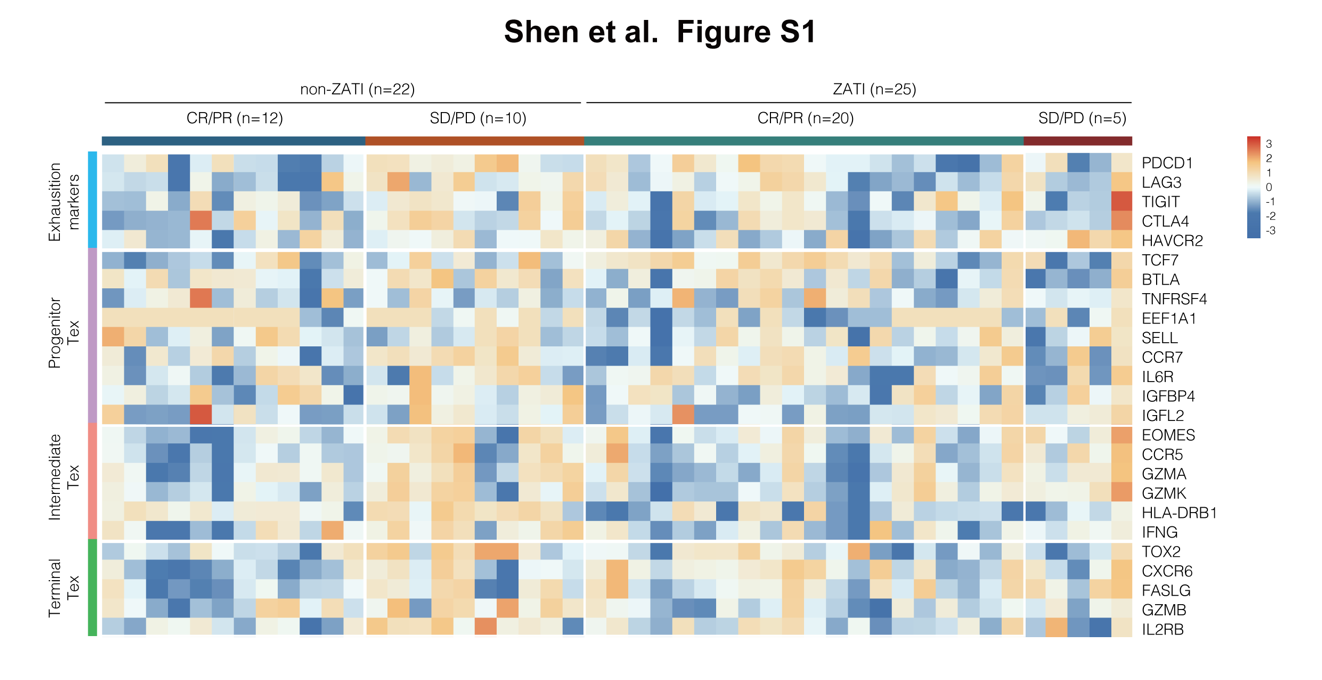


Shown are gene expressions for exhaustion markers and developmental stages of Tex cells measured by transcriptomic sequencing on available tumor biopsy specimens from patients in non-ZATI cohort (n = 22; 12 patients with CR/PR; 10 patients with SD/PD) and ZATI cohort (n = 25; 20 patients with CR/PR; 5 patients with SD/PD).

**Supplementary Figure S6. Pathway visualization for selected genes contributing to the KEGG annotation.**

**
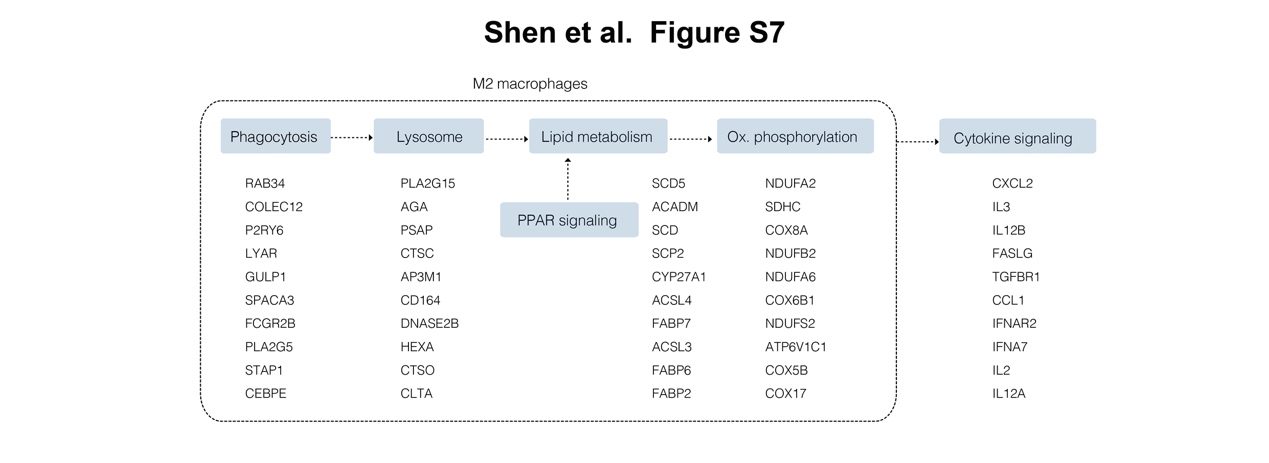
**

Shown are the genes contributing most to the enrichment score in the indicated KEGG pathways.
